# Membrane association of the ATG8 conjugation machinery emerges as a key regulatory feature for autophagosome biogenesis

**DOI:** 10.1002/1873-3468.14676

**Published:** 2023-06-10

**Authors:** Sharon A. Tooze, Wenxin Zhang, Gianmarco Lazzeri, Deepanshi Gahlot, Lipi Thukral, Roberto Covino, Taki Nishimura

**Affiliations:** ^1^ Molecular Cell Biology of Autophagy Laboratory The Francis Crick Institute London UK; ^2^ Frankfurt Institute for Advanced Studies Germany; ^3^ CSIR‐Institute of Genomics and Integrative Biology New Delhi India; ^4^ Academy of Scientific and Innovative Research (AcSIR) Ghaziabad India; ^5^ PRESTO, Japan Science and Technology Agency Tokyo Japan; ^6^ Department of Biochemistry and Molecular Biology, Graduate School of Medicine The University of Tokyo Japan

**Keywords:** amphipathic α‐helix, ATG3, ATG8 lipidation, autophagosome size, autophagy, *cis*‐membrane association, MD simulation, membrane expansion

## Abstract

Autophagy is a highly conserved intracellular pathway that is essential for survival in all eukaryotes. In healthy cells, autophagy is used to remove damaged intracellular components, which can be as simple as unfolded proteins or as complex as whole mitochondria. Once the damaged component is captured, the autophagosome engulfs it and closes, isolating the content from the cytoplasm. The autophagosome then fuses with the late endosome and/or lysosome to deliver its content to the lysosome for degradation. Formation of the autophagosome, sequestration or capture of content, and closure all require the ATG proteins, which constitute the essential core autophagy protein machinery. This brief ‘nutshell’ will highlight recent data revealing the importance of small membrane‐associated domains in the ATG proteins. In particular, recent findings from two parallel studies reveal the unexpected key role of α‐helical structures in the ATG8 conjugation machinery and ATG8s. These studies illustrate how unique membrane association modules can control the formation of autophagosomes.

## Graphical abstract

During the formation of autophagosomes, the phagophore membrane expands rapidly and is decorated by lipidated ATG8s, LC3 and GABARAP. The amphipathic helix of ATG3, the ubiquitin‐like E2 enzyme, has unique properties that catalyse the conjugation of ATG8 to phosphatidylethanolamine (PE). Further membrane expansion occurs by interaction in *cis* when the N‐terminus of the lipidated ATG8 inserts into the membrane.

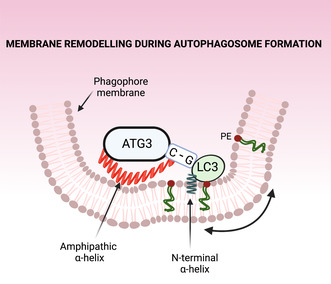

## Abbreviations

**AH**, amphipathic α‐helix

**ALPS**, amphipathic lipid packing sensor

**ATG**, autophagy related

**BATS**, Barkor/ATG14(L) autophagosome‐targeting sequence

**CLEM**, correlative light and electron microscopy

**Cryo‐CLXM**, cryo‐soft X‐ray tomography

**KO**, knock‐out

**LDS**, LIR docking site

**LIR**, LC3‐interacting region

**PI3KC3‐C1**, class I PtdIns‐3 kinase complex I

**PtdEtn**, phosphatidylethanolamine

**PtdIns 4‐P**, PtdIns 4‐phosphate

**PtdIns**, phosphatidylinositol

Formation of the autophagosome, sequestration or capture of content, and closure all require the ATG proteins, which act together in an efficient process set up as a hierarchy of complementary activities that are highly conserved [1]. Autophagy is upregulated by external stress conditions, such as nutrient deprivation, UV damage, and hypoxia. In long‐lived cells such as neurons, health and survival are dependent on autophagy [2], in particular selective forms of autophagy such as aggrephagy (removal of protein aggregates), mitophagy (removal of mitochondria) and ERphagy (removal of ER). In general, the role of autophagy, in particular selective autophagy, in human diseases such as neurodegeneration and cancer is complex [3] and will not be discussed here.

Initially identified in yeast through genetic screens, there are about 15 core ATG genes in mammalian cells (for comprehensive reviews, see Refs [4, 5]). Briefly, the initiation of autophagy begins with the formation of a phagophore, a double membrane with a curved cisterna, which expands, captures content (cargo) and closes, becoming an autophagosome. The phagophore and autophagosome are well described in morphological studies by many techniques, including confocal imaging, transmission electron microscopy, correlative light and electron microscopy (CLEM), and cryo‐soft X‐ray tomography (Cryo‐CLXM) [6, 7, 8, 9, 10]. Visualisation of autophagosome formation has largely relied on the detection of the lipidated tagged‐ATG8s both *in vitro* and *in vivo* experiments, although other peripheral membrane‐associated ATGs (e.g. ULK1, ATG13, Beclin1, WIPI2, ATG5, ATG16L1) can be detected to allow the different stages of autophagosome formation to be revealed.

**Fig. 1:**
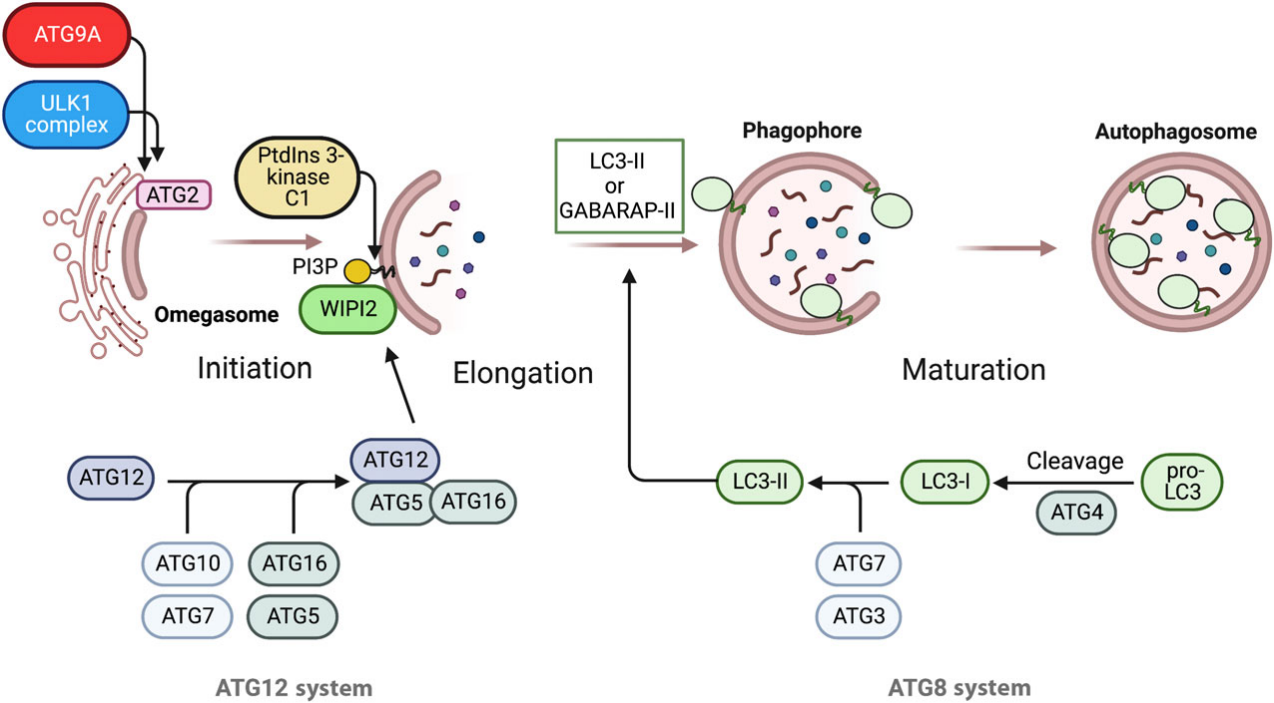
The autophagy pathway in mammalian cells. The formation of autophagosomes starts with the delivery of ATG9A in vesicles to the initiation site. Both ULK1/2 and ATG2 are also required at this initial step to form the omegasome. Recruitment of the class III PtdIns‐3 kinase (PI3KC3‐C1) produces PtdIns3P, which recruits WIPI2. Finally, WIPI2 recruits the E3‐like complex ATG5‐12‐16L1. The ubiquitin‐like conjugation systems required for lipidation of the ATG8s are shown.

The composition of the initiating membrane, called the omegasome [11], which precedes the phagophore, is not precisely known, but its formation is driven by lipid synthesis in the endoplasmic reticulum (ER) [12, 13] and the transfer of lipids through the ATG2A/B proteins, which are equilibrated through the activity of the lipid scramblase ATG9A [14, 15]. The consensus view is that the omegasome and phagophore membranes are similar to the ER, containing proportionally more phosphatidylethanolamine (PtdEtn) and phosphatidylinositol (PtdIns) than the plasma membrane [16]. In yeast, Atg9 vesicles have been shown to nucleate a membrane around cargo, which can expand to become an autophagosome [17]. In mammalian cells, the picture is more complex. Vesicles containing the lipid scramblase ATG9A are thought to either provide a seed membrane (similar to the yeast system [18]) or contribute significantly to the protein composition to alter the lipid composition of the phagophore membrane (Fig. 1), perhaps using a kiss‐and‐run mechanism [15, 19].

The central autophagy kinase complex ULK1/2 plays a key role in orchestrating these early events, in particular the trafficking of ATG9A [7]. Recruitment of ATG13, a subunit of ULK1, to the initiating membrane [20] occurs by binding PtdIns 4‐Phosphate (PtdIns 4‐P). PtdIns 4‐P is produced by PtdIns‐4 Kinase IIIβ (PI4KB), which is delivered by ATG9A vesicles [19]. Additional modification of the PtdIns in the early stages of the omegasome and later of the forming phagophore by the class I PtdIns‐3 kinase complex I (PI3KC3‐C1) occurs downstream of the key autophagy kinase, ULK1 [21]. ATG14, the class I‐specific component of the PI3KC3‐C1 complex, localises to the ER [20], and binds through the BATS domain [22], which recognises PtdIns in higher curvature membrane domains [23, 24]. The BATS domain in ATG14 contains an amphipathic lipid packing sensor (ALPS) motif, which senses the packing of lipids in the bilayer [25]. ALPS motifs are considered long amphipathic α‐helixes. An amphipathic α‐helix (AH) is also found in VPS34, another subunit of the PI3KC3.

Acquisition of PtdIns3P on the omegasome and phagophore recruits autophagy‐specific effectors such as DFCP‐1 [11] and WIPI2 [26]. WIPI2 then recruits the E3‐like complex ATG12—ATG5‐ATG16L1 to the phagophore, which directs the association of ATG8s with membranes through covalent modification by PtdEtn (lipidation). Interestingly, ATG16L1 contains two regions that allow membrane association, one being an amphipathic α‐helix [27].

Lipidation mediated by the ubiquitin‐like conjugation pathway (see below) is critical for phagophore expansion, autophagosome growth and closure in both non‐selective and selective autophagy. During and after lipidation, discrete perturbations of the membrane bilayer mediated by the conjugation pathway proteins and ATG8 occur due to the intrinsic association of these proteins with the bilayer. Recent findings about the mechanism of membrane association of ATG3 [28] and the ATG8 proteins [29], described below, have contributed to a further understanding of how protein‐membrane interactions influence autophagosome formation.

## The ATG8 family

In yeast, there is a single Atg8, while in mammalian cells, there are six ATG8s, three in the LC3 family and three in the GABARAP family [30, 31]. ATG8s are small ubiquitin‐like proteins that have essential roles in autophagy and unique functionality. ATG8s have a ubiquitin core and a unique N‐terminal domain formed by two α‐helices. Most critical for selective autophagy and the best studied function of the ATG8s is their ability to bind cargo containing a LIR (LC3‐interacting region) motif (for review, see Ref. [31]). The LIR motif (called AIM, Atg8‐interacting motif in yeast) in the selective autophagy receptors (SARs) and autophagy adaptors binds to the LDS (LIR docking site) on the ATG8s. The LDS is formed by two hydrophobic pockets created by the N‐terminal helices of the ATG8s and residues in the ubiquitin core. Briefly, the LIR motif in SARs and adaptors enable cargo recruitment, but is also present in proteins which are recruited to the outer surface of autophagosome and there, for example, facilitate autophagosome movement (FYCO1) and fusion with lysosomes. Certain ATG proteins such as ULK complex members also contain LIR motifs and bind to ATG8s (for review, see Ref. [32]). One consensus that has emerged is that the LC3 family functions to recruit cargo to the inner membrane of the cup‐shaped phagophore, while the GABARAP family acts at the highly curved rims and outer surface of the phagophore. In this regard, GABARAPs are implicated in the closure of the autophagosome as well as post‐closure events such as transport to and fusion with lysosomes; for review, see Ref. [33].

## ATG8 conjugation and the role of ATG3 membrane association

**Fig. 2:**
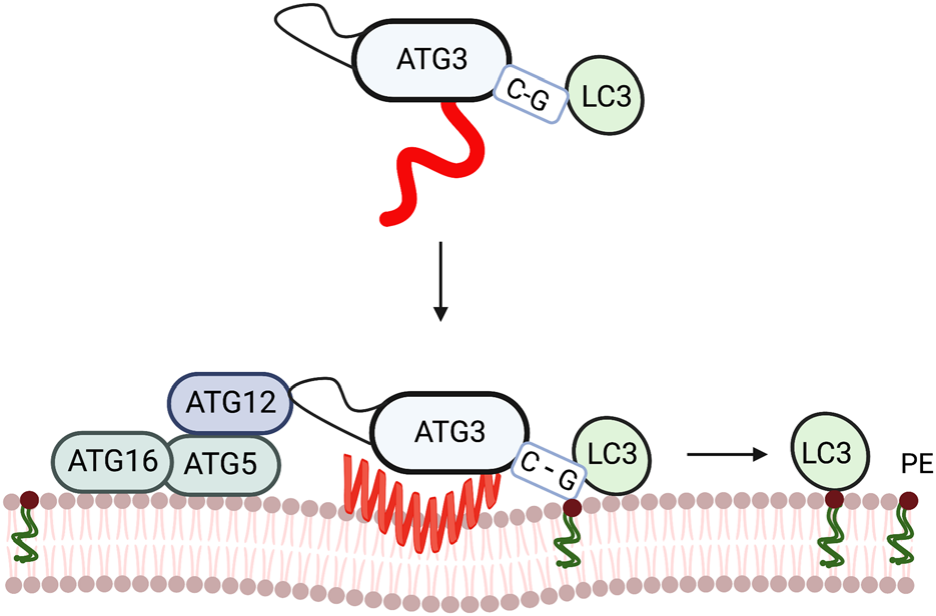
The association of the AH in ATG3 with the membrane during conjugation. The N‐terminus of ATG3 contains a highly conserved AH. This AH has unique properties that facilitate the catalytic activity of ATG3 and thus the lipidation of LC3/GABARAP.

LC3s and GABARAPs undergo identical modifications leading to lipidation of the C‐terminal glycine (Fig. 2). This ubiquitin‐like conjugation pathway was first discovered in yeast and is conserved in eukaryotes [34]. The initial conversion to an activated ATG8 is performed by the ATG4 family of endoproteases, which expose a C‐terminal glycine. Subsequently, in ATP‐dependent reactions, the glycine in ATG8 is conjugated to ATG7, the E1‐like protein, followed by the transfer of ATG8s to E2, ATG3. Next, the targeting of the ATG3‐ATG8 complex to membranes is facilitated by the E3‐like complex, ATG12—ATG5‐ATG16. Once in proximity to the membrane and the ATG12—ATG5‐ATG16 complex, ATG3 binds to ATG12 and catalyses the transfer of LC3 to PtdEtn, allowing successful conjugation (or lipidation) of the ATG8s to the phagophore and autophagosome. Visualisation of autophagosome formation has largely relied on the detection of the lipidated ATG8s on the membrane, although many studies have also visualised single components of the E3 complex, ATG5, ATG12 or ATG16L1.

ATG3 contains an N‐terminal amphipathic α‐helix (ATG3_AH_) that enables membrane association [35]. As detailed above, amphipathic helixes (AH), which are found in a number of ATG proteins, are also widespread throughout the proteome [36] and are key lipid‐binding modules. Detailed mechanistic insight into how and where AHs associate with the lipid bilayer is just emerging using techniques such as NMR and molecular dynamics simulations. *In vivo* and *in vitro* evidence revealed that the ATG3_AH_ is key to target membrane regions with specific curvatures [35]. However, the same study revealed that, in addition to curvature sensing, ATG3_AH_ also plays a key role in regulating the catalytic site of ATG3 allosterically. Key questions remained. How does the ATG3‐ATG8 conjugate specifically associate with phagophore and autophagosome membranes? Does this specificity go beyond curvature? And is the specificity driven by the properties of the ATG3_AH_? Additionally, how is the ATG3_AH_ mediating information from the membrane proximal region to its catalytic site.

These questions were addressed by Nishimura *et al*. [28]. A chimeric domain‐swap of AHs from ATG proteins, including VPS34, replacing the ATG3_AH_ demonstrated that lipidation of LC3 requires the ATG3_AH_. Further phylogenetic, biochemical and morphological analysis confirmed a unique biophysical and biochemical fingerprint of the N‐terminal AH of ATG3. Surprisingly, unlike many other AHs, the fingerprint of ATG3_AH_ is unique amongst AHs. Phylogenetic analysis of ATG3_AH_ and unsupervised machine learning of over 1800 AHs indicated that the N‐terminal ATG3_AH_ fingerprint was finely tuned, characterised by low hydrophobicity and a few bulky hydrophobic amino acids. These characteristics are critical for efficient LC3 lipidation.

Molecular dynamics (MD) simulations revealed that the dynamic organisation of ATG3_AH_ in the membrane is critical for LC3‐lipidation. The low hydrophobicity of the ATG3_AH_ ensured efficient positioning of the catalytic site, allowing LC3 to access the headgroup of PtdEtn while ensuring dynamic association and dissociation with the lipid bilayer. While catalysis required ATG3 binding to ATG12, the discovery of the unique AH properties supports the hypothesis that a transient intermediate between the PtdEtn headgroup and the glycine residue of LC3, allowing release of ATG3 from the conjugate and membrane, is essential for lipidation (Fig. 2).

Simple models based on the assumption that curvature sensing is the only function of AHs in targeting membranes would predict that a stronger association with the membrane would be beneficial. However, mutagenesis of the ATG3_AH_, for instance, substitution of the low hydrophobic residues in the AH with bulky hydrophobic residues, leads to stalling of autophagosome progression, retention of ATG3 on ATG5‐positive structures on the membrane, and inhibition of lipidation. MD simulations showed that the additional hydrophobic residues alter the dynamics not only of the AH in the membrane but more globally of the whole complex, decreasing the proximity of the catalytic site to the lipids [28]. This result illustrates how interactions between residues in the membrane‐targeting region and the membrane are fine‐tuned and modulate the structural dynamics of the whole complex. Additionally, these interactions can also be modified by changing the membrane lipid composition. Taken together, these observations reveal a new layer of complexity in understanding the regulation of autophagy‐related proteins.

The marginally stable association of the ATG3_AH_ was unexpected and distinct from that of other AHs in the ATG family, in particular the AH of ATG14, which is most similar in composition to ATG3. The ATG14_AH_ cannot replace the AH in ATG3, supporting the notion that the ATG3_AH_ is not exclusively functioning as a membrane‐curvature sensor but also as a catalytic regulator of lipidation and autophagosome formation. Future work is required to further elucidate the mechanism.

## Autophagosome expansion and the role of the N‐terminus of ATG8s

Downstream of ATG3 and ATG8 lipidation, and cargo recruitment, is an additional fundamental role of the ATG8s in driving the morphological transition of the double membrane sheet created by the ER‐omegasome platform into a phagophore and eventually a spherical closed compartment with an outer and inner membrane. This transition has been mathematically described by Knorr and colleagues [37]. In this mathematical model, the membrane is shaped by a protein condensate formed into a droplet by the cargo SQSTM1/p62. Regardless of how the membrane is shaped (cargo dependent or independent), there is a requirement for membrane expansion and closure. These events require perturbation of the lipid sheet at the highly curved edges, as well as changes in the relatively flat outer convex surface, and negatively curved inner concave membrane.

Autophagosome size has been shown to depend on Atg8s in yeast [38] and *Caenorhabditis elegans* during embryogenesis [39]. A theoretical model predicts that edge stabilisation by membrane‐shaping proteins, including ATG proteins, can be a key factor for autophagosome size regulation [40]. In mammalian cells, knock out of all ATG8 proteins results in smaller autophagosomes, though autophagosome formation is still maintained [41]. Historically, the ATG8s were first considered to function in fusion by bringing together two separate membranes in a *trans* reaction, in which the ATG8 C‐terminal glycine is anchored on one membrane and its N‐terminal helices associate with the opposing membrane [42, 43]. More recently, a *cis* activity has been described, whereby the insertion of two aromatic residues (F77/F79) present in the ubiquitin core in yeast Atg8 causes the membrane perturbation and the formation of tubulovesicular compartments, which contribute to autophagosome biogenesis [44]. However, these data do not fully explain how ATG8 controls the size of the autophagosome.

**Fig. 3:**
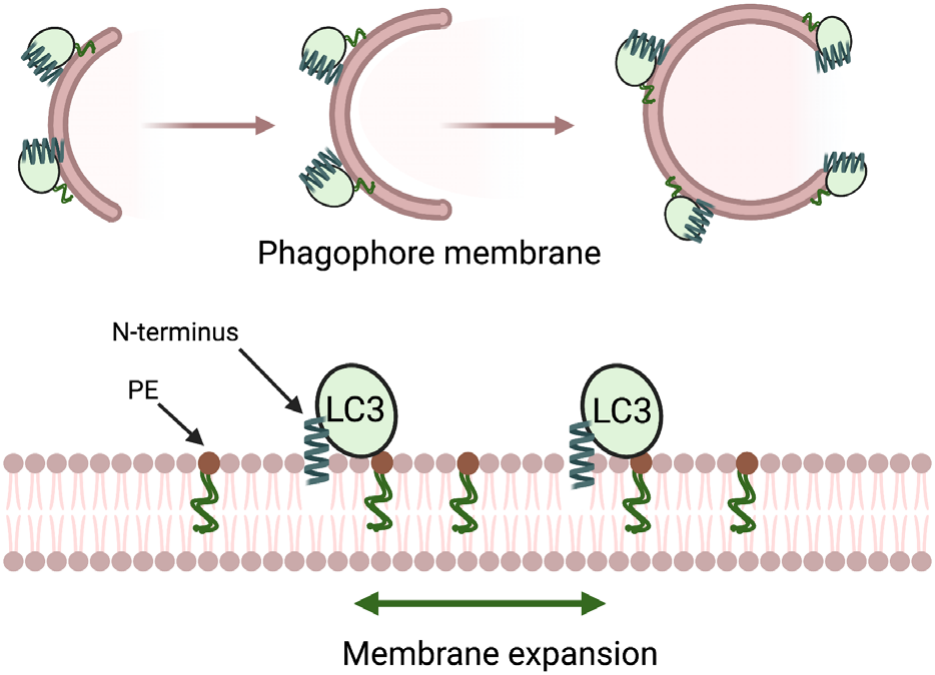
Membrane expansion driven by the N‐terminal helix of ATG8s (LC3s and GABARAPs). The N‐terminal domain of LC3/GABARAP associates in *cis* with the phagophore membrane, driving membrane expansion. The *cis* association is required for formation of normal‐sized autophagosomes, and functions independently of autophagy cargo.

Changes in the size of an autophagosome should occur in parallel with the shape change attributed to the F77 and F79 aromatic residues. Considering the structure of the ATG8s in comparison to the structure of ubiquitin, it is apparent that one distinct feature is the extended α‐helices (α1, α2) in the N‐terminus of the ATG8 family appended to the ubiquitin core. Using newly developed real‐time assays [29] and fully exploiting the ability to reconstitute lipidation of ATG8s *in vitro*, the role of the N‐terminal α1‐helix was probed to determine if it was involved in autophagosome growth. *In vitro* lipidation of the ATG8s can be reconstituted using purified components (ATG7, ATG3, ATG12—ATG5‐ATG16L1), ATP and liposomes containing PtdEtn. Purified ATG8s can be tagged at the N‐terminus with NBD, an environmentally sensitive fluorescent molecule, allowing detection of lipidation and hence providing a tool to test the role of the α1‐helix of the LC3s and GABARAPs. Combining these approaches with all‐atom MD simulations, key residues in α1‐helix of both LC3 and GABARAP were identified, which were inserted into the same lipid bilayer where their C‐terminal glycine is anchored [29] with additional residues in the ubiquitin core (a *cis* interaction model), see Fig. 3. Notable differences in the ability of LC3s and GABARAP to interact with the conjugation machinery and membranes were revealed using mutations in the N‐terminus and core *in vitro*, which supports a discrimination of the LC3 and GABARAP family members in both their ability to interact with the conjugation machinery and hydrophobic lipid environments.

The unique properties of GABARAP in autophagy were studied using the ATG8 knockout cell model, Hela Hexa KO [41], in which all six ATG8s (LC3 and GABARAPs) are absent. The non‐tagged GABARAP constructs, wild‐type or with mutations in the membrane associated N‐terminal and core residues, were still lipidated, showing that these mutations did not affect the conjugation pathway. Furthermore, N‐terminal deletions and core mutants were able to rescue p62 degradation as efficiently as wild‐type GABARAP, indicating that the membrane association residues in N‐terminus and the core were not involved in cargo degradation. Using CLEM to determine the morphology, in particular the size of the autophagosomes, in the ATG8 Hexa KO after rescue with GABARAP mutants, the detectable autophagosomes were significantly reduced in size and volume, while the wild‐type GABARAP restored the autophagosomes to a normal size [29]. These data can be interpreted to show that the N‐terminus of the GABARAPs, by inserting in a *cis* manner into the membrane, has the ability to drive the expansion and growth of the autophagosome. The mechanism of expansion needs further exploration but is likely related to lipid packing defects observed *in vitro* in the real‐time assays.

## Conclusions

α‐Helices can anchor the protein to the membrane, ensuring its proper localisation and orientation [20, 45]. Furthermore, amphipathic α‐helices can also function as sensors or modulators of the membrane curvature [46]. In this brief nutshell, two modes of association of ATG proteins with membranes have been described. One is a classical amphipathic α‐helix, which allows association of ATG3 with membranes, while the second is a small helix of 11 amino acids at the N‐terminus of ATG8s. The consequence of disabling these membrane association regions is a loss of control over autophagosome biogenesis.
